# Laparoscopic Diagnosis of an Incarcerated Ileal Conduit in an Inguinal Hernia: A Case Report

**DOI:** 10.7759/cureus.104791

**Published:** 2026-03-06

**Authors:** Takumi Ishikawa, Ken Nakata, Kaoruko Iwasa, Tetsuya Murakawa, Yuki Nishimura, Makoto Ishii, Takahiro Imanaka, Masao Kobayashi, Yutaka Ono

**Affiliations:** 1 Department of Urology, Higashiosaka City Medical Center, Higashiosaka, JPN; 2 Department of Surgery, Higashiosaka City Medical Center, Higashiosaka, JPN

**Keywords:** ileal conduit, incarcerated inguinal hernia, obstructed inguinal hernia, obstructive pyelonephritis, radical cystectomy, radical cystectomy with an ileal conduit, sliding inguinal hernia

## Abstract

Ileal conduit creation is a common urinary diversion after radical cystectomy, though complications such as obstruction can occur. We report a rare case of an 80-year-old male with recurrent obstructive pyelonephritis due to an incarcerated ileal conduit in an inguinal hernia.

An 80-year-old male, 27 years post-total cystectomy, presented with recurrent fever due to obstructive pyelonephritis. For three years, he suffered from repeated urinary tract obstructions attributed to kinking of the ileal conduit, requiring multiple hospitalizations and catheter placements, despite regular follow-up at our institution and affiliated hospitals. Laparoscopic exploration, initially planned for conduit repair, unexpectedly revealed the ileal conduit incarcerated within an inguinal hernia, establishing the definitive diagnosis.

Although rare, incarceration of the ileal conduit should be considered in the differential diagnosis of urinary tract obstruction in patients with an ileal conduit, particularly when the cause of obstruction is unclear.

## Introduction

Ileal conduit creation is a common urinary diversion procedure following radical cystectomy. While generally safe, complications such as urinary tract obstruction can occur. Typical causes of late urinary tract obstruction include ureteroileal anastomotic stricture, stomal or conduit stenosis, and urolithiasis. In contrast, we present a case of an 80-year-old male who developed recurrent obstructive pyelonephritis due to an incarcerated ileal conduit in an inguinal hernia, a condition rarely reported in the literature with typically reported intervals ranging from months to over a decade post-surgery [1-3]. This case underscores the diagnostic challenges and the role of laparoscopic exploration in identifying such unusual complications.

## Case presentation

Patient information

The patient was an 80-year-old male whose chief complaint was fever. His medical history included pulmonary emphysema and chronic kidney disease. He was not taking any regular medications.

History of present illness

**Figure 1:**
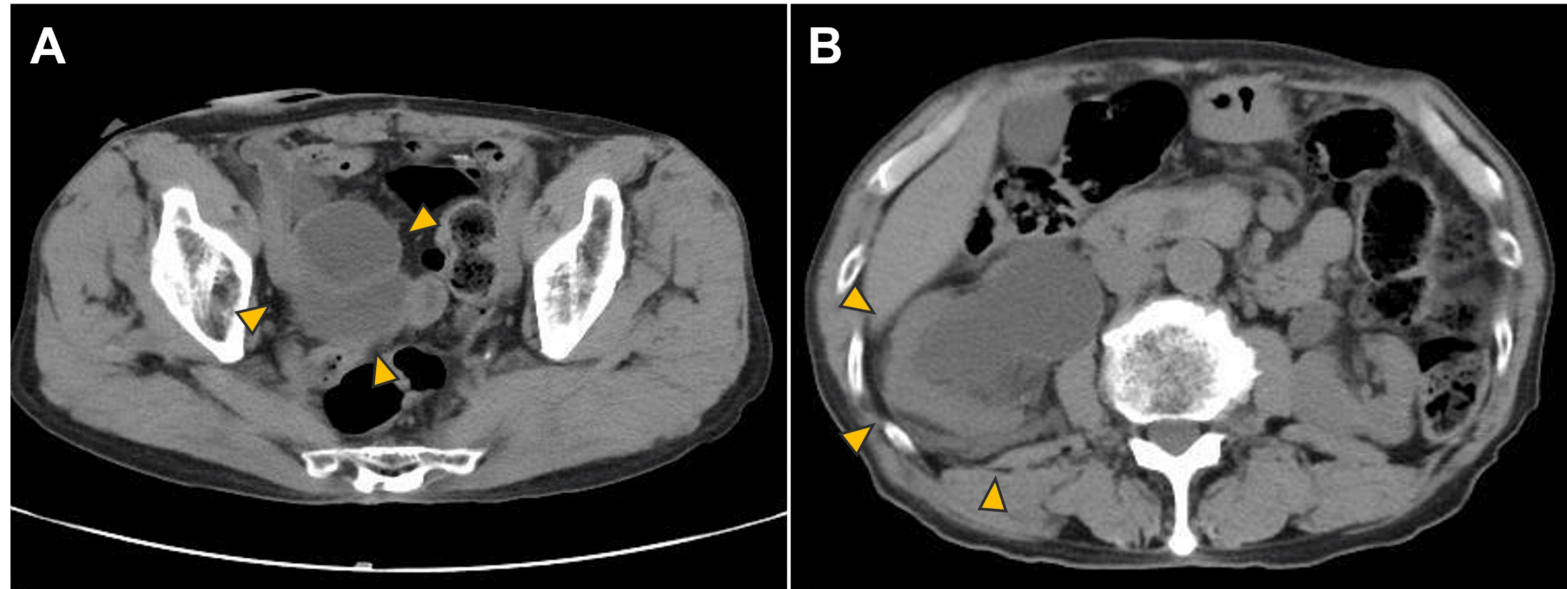
Computed tomography (CT) findings at the onset of symptoms (three years prior) Axial CT images were obtained three years prior to the current presentation. (A) Image showing the dilated ileal conduit (orange arrowheads). (B) Image demonstrating right hydronephrosis (orange arrowheads). Although these findings indicated urinary tract obstruction, the definitive cause was not identified at this stage.

Twenty-seven years prior to the current presentation, the patient underwent open radical cystectomy and ileal conduit creation for bladder cancer. Three years prior, he developed obstructive pyelonephritis due to kinking of the ileal conduit, causing urinary tract obstruction (Figure 1).

**Figure 2:**
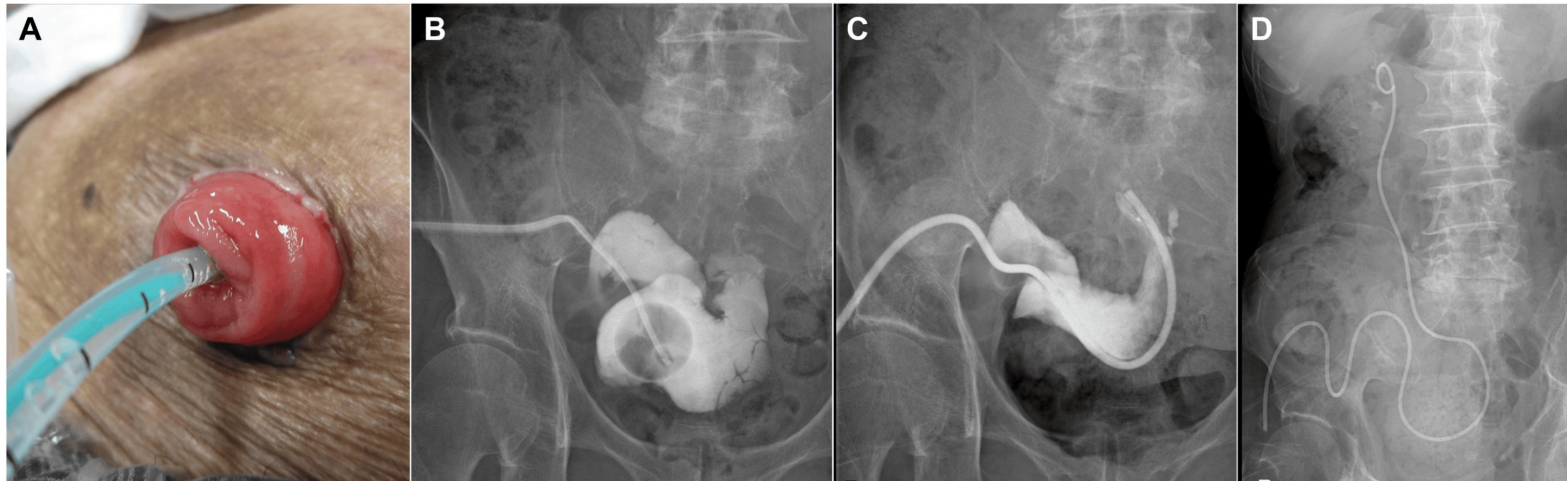
History of catheter management for recurrent obstruction (A) Appearance of the ileal conduit stoma with an indwelling renal pelvis balloon catheter. (B) Abdominal radiograph showing the indwelling renal pelvis balloon catheter. (C) Abdominal radiograph showing a Nelaton catheter placed in the ileal conduit. (D) Abdominal radiograph showing a single-J (SJ) catheter placed in the right renal pelvis.

Over the subsequent three years, he was under regular clinical follow-up at our institution and affiliated hospitals. Various attempts were made to manage the obstruction, including placement of a renal pelvis balloon catheter and a Nelaton catheter into the ileal conduit (Figure 2A-2C). Single-J (SJ) and double-J (DJ) catheters were also placed into the renal pelvis and ureter (Figure 2D).

However, due to spontaneous dislodgement or inability to replace the catheters by blind technique, management became difficult.

Each time the catheter dislodged, re-insertion into the ileal conduit required cystoscopic guidance to pass a guidewire to the conduit's blind end before successful catheter placement. This difficult management led to nine repeated hospitalizations and discharges. During this period, the patient did not report groin pain, visible bulging, or other symptoms suggestive of an inguinal hernia on physical examination. These recurrent admissions prompted multidisciplinary discussions involving urology, general surgery, and radiology teams. The patient was advised to undergo ileal conduit repair and was referred to the Department of Surgery.

Physical examination and laboratory findings on admission

**Figure 3:**
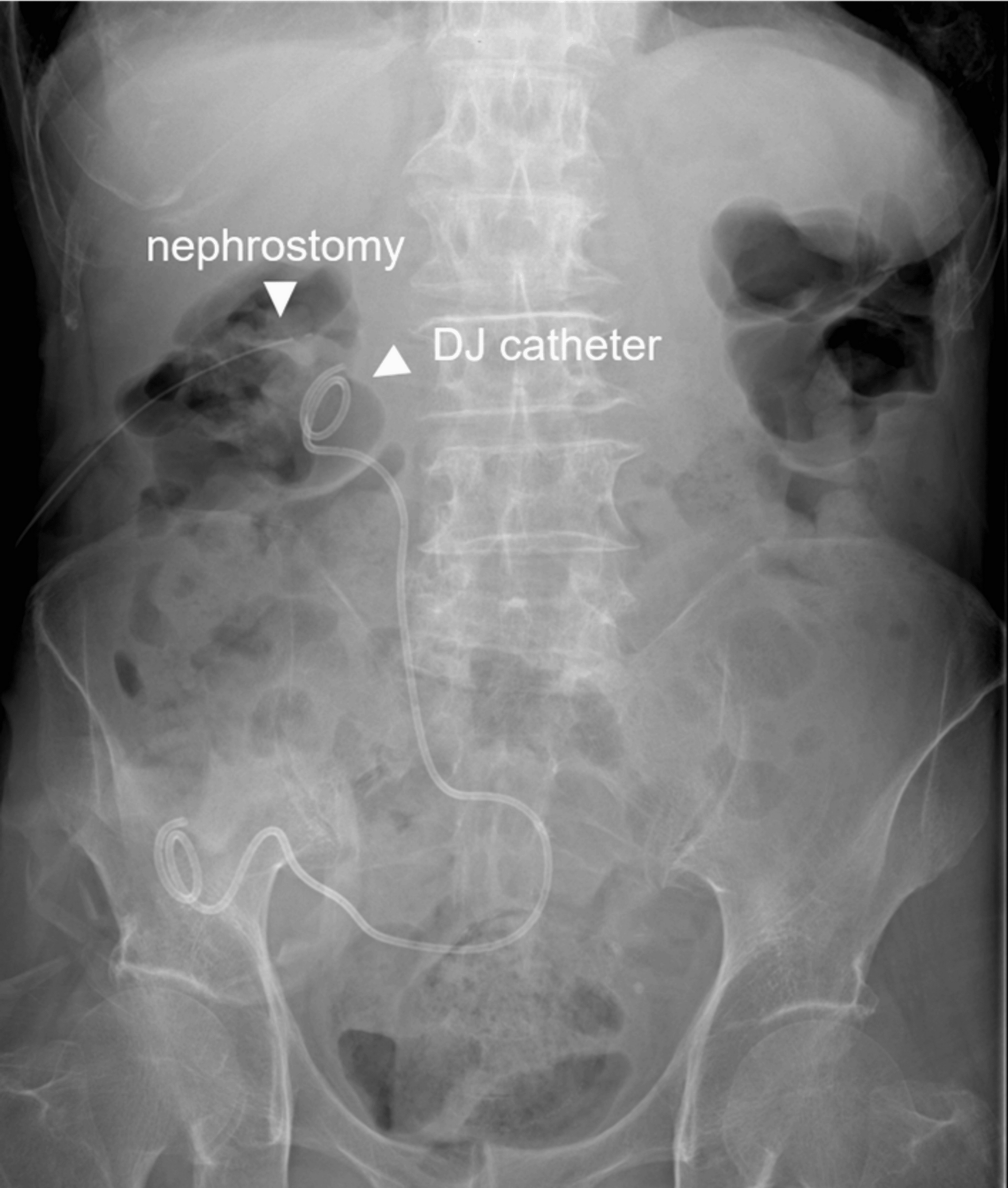
A plain abdominal radiograph taken on admission shows a right nephrostomy tube and a right double-J (DJ) catheter in place

On admission, the patient's height was 152 cm, and weight was 42 kg. Physical examination revealed a right nephrostomy and a right DJ catheter in place (Figure 3).

Laboratory findings on admission are summarized in Table 1. Notably, elevated serum creatinine (3.64 mg/dL) and urea nitrogen (62.4 mg/dL) levels were observed, reflecting renal impairment secondary to the recurrent urinary tract obstruction.

**Table 1:** Laboratory findings on admission WBC: white blood cell; RBC: red blood cell; Hb: hemoglobin; Ht: hematocrit; PLT: platelet; UN: urea nitrogen; Cre: creatinine; AST: aspartate aminotransferase; ALT: alanine aminotransferase; CRP: C-reactive protein; Alb: albumin

Parameter	Result	Unit	Normal Range
Hematology			
WBC	5140	/μL	3500-9000
RBC	296	×10^4^/μL	430-570
Hb	9.8	g/dL	13.5-17.5
Ht	30.4	%	40-50
PLT	17.3	×10^4^/μL	15-40
Biochemistry			
Na	135	mEq/L	135-145
K	4.8	mEq/L	3.5-5.0
Cl	107	mEq/L	98-107
UN	62.4	mg/dL	8-20
Cre	3.64	mg/dL	0.6-1.1
AST	40	U/L	10-40
ALT	31	U/L	5-40
CRP	0.22	mg/dL	<0.3
Alb	3.5	g/dL	3.8-5.2

Surgical findings

**Figure 4:**
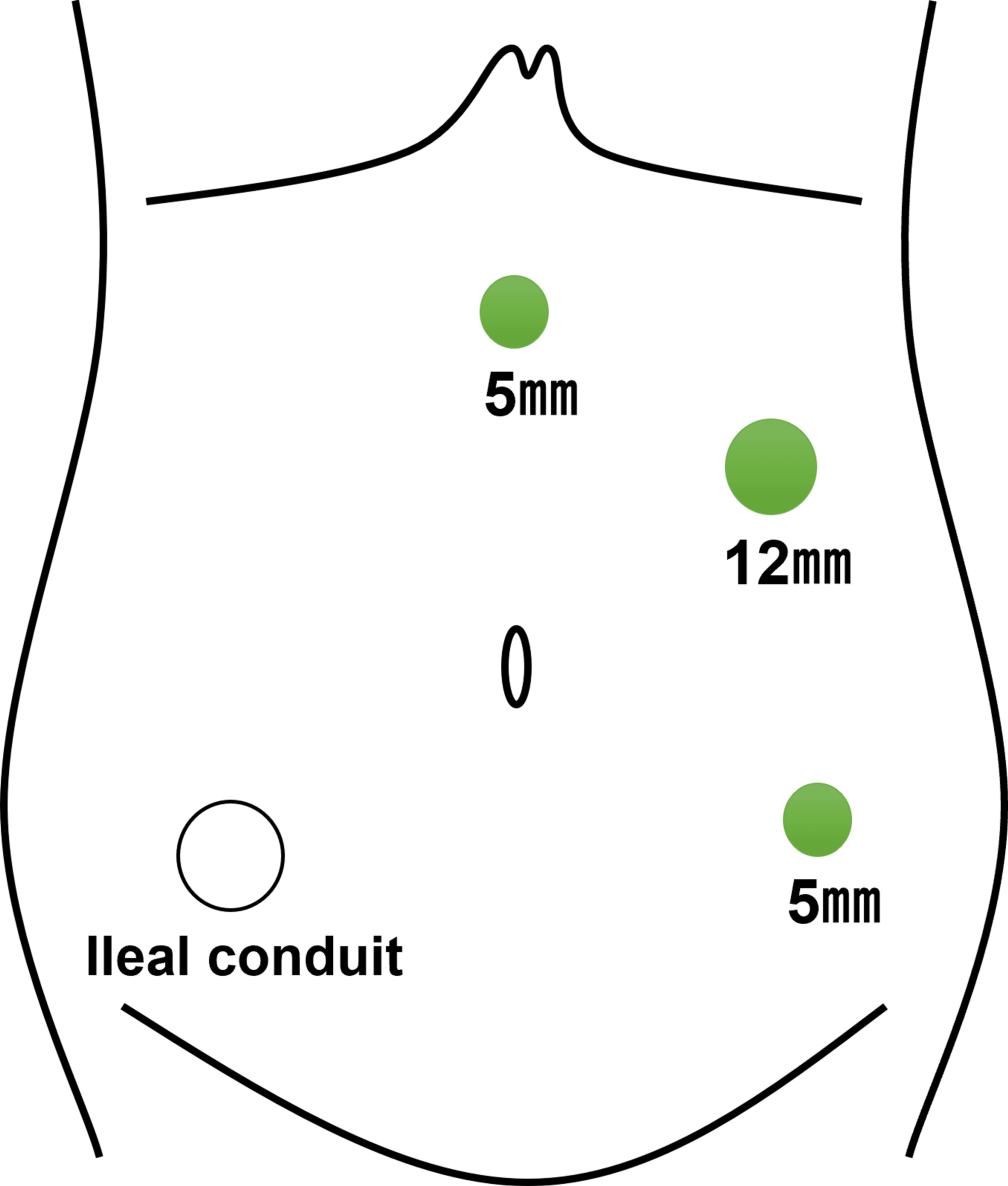
Schema of laparoscopic port placement A 12-mm camera port was placed on the left side of the abdomen. Two 5-mm working ports were positioned to encircle the ileal conduit, which was located in the right lower abdomen. Source: Illustration created by the authors using Microsoft PowerPoint (Microsoft Corp., Redmond, WA, USA).

Laparoscopic surgery was performed with the primary goal of shortening, fixing, or bypassing the ileal conduit, as kinking of the conduit was considered the main cause of obstruction. The ileal conduit was identified in the right lower abdomen. A 12-mm camera port and two 5-mm working ports were placed encircling the conduit (Figure 4).

Adhesions were not encountered during port placement.

**Figure 5:**
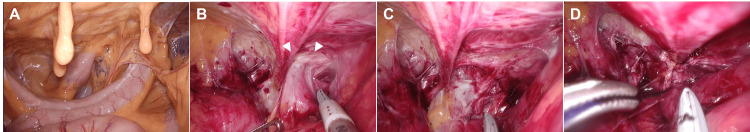
Intraoperative findings (A) Initial laparoscopic view showing adhesions between the small bowel and the anterior abdominal wall. (B) After adhesiolysis, the ileal conduit was unexpectedly found to be incarcerated within the right inguinal hernia orifice (arrowheads), establishing the definitive diagnosis. (C) View of the hernia defect after reducing the incarcerated conduit. (D) The hernia defect was closed using a running suture technique.

Initial laparoscopic inspection revealed adhesion of the small bowel, including the ileal conduit, to the anterior abdominal wall (Figure 5A). These adhesions were carefully dissected using monopolar scissors. During the adhesiolysis, the ileal conduit was unexpectedly found to be incarcerated within an inguinal hernia orifice (Figure 5B).

The incarceration of the ileal conduit within the inguinal hernia orifice established the definitive diagnosis, explaining the patient's history of chronic, recurrent, and refractory obstruction.

The incarcerated segment of the ileal conduit was gently reduced. Given the insufficient space around the hernia defect and the proximity of the ileal conduit, safe tension-free mesh placement was precluded due to the high risk of mesh infection. Furthermore, a totally extraperitoneal approach was deemed extremely difficult and unsafe because the preperitoneal space was presumed to be obliterated by severe adhesions and scarring from the previous open radical cystectomy. Therefore, primary suture repair of the hernia was elected. The defect was closed using a running suture technique (Figures 5C, 5D).

The total operative time was two hours and 47 minutes, and the estimated blood loss was 50 mL. The postoperative course was uneventful. The patient was discharged without complications and has shown no recurrence of ileal conduit obstruction or pyelonephritis to date.

## Discussion

Incarceration of the ileal conduit within an inguinal hernia is an extremely rare complication. To our knowledge, there have been no reported cases in Japan, and only three cases have been documented in the English literature [1-3]. Previous reports indicate varying intervals between cystectomy and the onset of incarceration. Young and Weston [1] reported a case occurring 15 months postoperatively, while Ramayya [2] and Green and Dodds [3] reported cases occurring 14 and 10 years after surgery, respectively. In contrast, our patient presented with this complication 27 years after the initial surgery. This latency period is exceptionally long compared to previous reports. Although long-term complications of ileal conduits are well-documented, they can typically occur even decades postoperatively [4]. A systematic review by Zhu et al. regarding radical prostatectomy demonstrated that the majority of postoperative inguinal hernias develop within the first two years [5]. The extremely late onset in our case, combined with the lack of apparent hernia symptoms, likely contributed to the delay in diagnosis and the failure to recognize the hernia as the cause of the recurrent obstruction.

Several anatomical risk factors for ileal conduit incarceration have been suggested, including a redundant conduit length, failure to peritonealize the proximal end, and lack of fixation of the conduit to the abdominal wall. In the case reported by Green and Dodds [3], incarceration occurred despite minimizing these risks, suggesting that the development of an inguinal hernia itself is a critical independent risk factor. Therefore, we advocate that if an inguinal hernia is identified in a patient with an ileal conduit, simultaneous repair should be strongly considered to prevent future incarceration.

The surgical approach to urinary diversion may also influence the incidence of this complication. At our institution, during the era of open radical cystectomy, we routinely performed peritonealization and fixation of the ileal conduit to the abdominal wall. However, with the transition to robot-assisted radical cystectomy (RARC), we have adopted extracorporeal urinary diversion [6]. Consequently, the conduit is neither peritonealized nor fixed to the abdominal wall in our current practice. Although minimally invasive techniques offer many benefits, the omission of these fixation steps could theoretically increase the mobility of the conduit. Similar complications, such as inguinal bladder hernias, have been reported in 1-4% of inguinal hernias [7], and rare cases of neobladder herniation have also been documented [8]. These findings highlight the importance of considering urinary tract involvement in inguinal hernias. We suggest that current shifts in surgical techniques might lead to an increased incidence of incarcerated ileal conduits in inguinal hernias in the future, warranting heightened awareness and vigilant follow-up.

**Figure 6:**
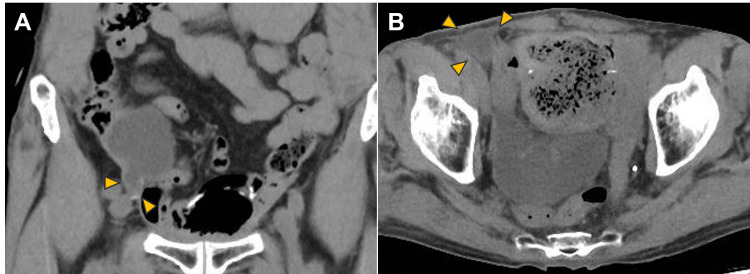
Retrospective review of preoperative CT scans (A) Coronal and (B) axial CT images showing the ileal conduit extending into the right inguinal canal (orange arrowheads). These subtle findings were indicative of incarceration but were initially overlooked due to the rarity of this complication.

Diagnosing this condition is challenging because the clinical presentation mimics simple stomal stenosis or uretero-ileal anastomotic stricture. In our case, retrospective review of CT images revealed subtle signs of the conduit entering the inguinal canal, which were initially overlooked (Figure 6).

Although CT is a key modality for evaluating complications after urinary diversion, interpretation can be challenging due to altered postoperative anatomy, requiring comprehensive knowledge of the surgical reconstruction [9]. This case underscores that in patients with unexplained or recurrent conduit obstruction, the possibility of herniation should be investigated, regardless of the time elapsed since surgery.

In this case, laparoscopic exploration was effective for both diagnosis and treatment. The international guidelines support the laparoscopic approach for groin hernias, highlighting its utility in evaluating the viability of incarcerated contents and minimizing wound complications [10].

Clinical clues such as recurrent obstruction without anastomotic stricture, difficulty maintaining catheter position, and conduit deviation toward the groin on CT should strongly raise suspicion for hernia incarceration. As a single-case report with a limited follow-up duration, these findings cannot be generalized to establish causality or incidence. Nevertheless, this case highlights the critical need for increased awareness and careful consideration of preventive surgical techniques.

## Conclusions

We report a rare case of recurrent obstructive pyelonephritis secondary to urinary tract obstruction caused by an incarcerated ileal conduit within an inguinal hernia, which posed significant diagnostic and therapeutic challenges. This complication is extremely rare but highlights the critical need for increased awareness and careful consideration of preventive surgical techniques, particularly given the shift toward evolving procedures such as robotic radical cystectomy with extracorporeal urinary diversion.
